# Analysis of catastrophic costs incurred by patients with multidrug-resistant tuberculosis in an outpatient clinic in the state of Rio de Janeiro

**DOI:** 10.1590/0037-8682-0148-2023

**Published:** 2023-10-30

**Authors:** Regielle Luiza de Medeiros, Adriana da Silva Rezende Moreira, Ana Carolina de Oliveira Jeronymo Neves, Viviane de Jesus Leite, Isabela Neves de Almeida, Fernanda Carvalho de Queiroz Mello, Afrânio Kritski

**Affiliations:** 1 Universidade Federal do Rio de Janeiro, Programa Acadêmico de Tuberculose, Escola de Medicina, Rio de Janeiro, RJ, Brasil.; 2 Universidade Federal do Rio de Janeiro, Programa de Pós-Graduação em Ensino de Matemática, Instituto de Matemática, Rio de Janeiro, RJ, Brasil.; 3 Universidade Federal de Ouro Preto, Programa de Pós-Graduação em Biotecnologia, Núcleo de Pesquisa em Ciências Biológicas, Ouro Preto, MG, Brasil.; 4 Universidade Federal do Rio de Janeiro, Instituto de Doenças do Tórax, Rio de Janeiro, RJ, Brasil.

**Keywords:** Multidrug-resistant tuberculosis, Costs, Direct costs, Indirect costs

## Abstract

**Background:**

Multidrug-resistant tuberculosis (MDR-TB) is a serious global public health concern associated with social vulnerability. In Brazil, the Unified Health System (SUS, Portuguese) provides free diagnosis and treatment for MDR-TB; however, other expenses may still be incurred for patients and their families which, according to the World Health Organization (WHO), can be catastrophic when these costs surpass 20.0% of the annual household income. This study aimed to assess the extent of catastrophic costs related to the diagnostic and therapeutic aspects of MDR-TB among patients receiving care at an outpatient clinic in Rio de Janeiro.

**Methods:**

This prospective study used convenience sampling from July 2019 to June 2021. Data regarding direct and indirect costs were collected using a standardized questionnaire endorsed by the WHO. To analyze any impoverishment occurred from MDR-TB, a threshold established by the Brazilian Institute of Geography and Statistics for 2019 and 2020 of US$ 79,562 and US$ 94,5273, respectively, was applied. Descriptive statistics were used for data analysis, including mean; standard deviation; variation coefficient; median; and maximum, minimum, and interquartile ranges.

**Results:**

A total of 65 patients were interviewed. Among the participants, 73.8% experienced catastrophic costs, with indirect costs exerting the most significant impact (median: US$ 3,825.9), in contrast to direct costs (median: US$ 542.7). When comparing the periods before and after diagnosis, the prevalence of poverty increased from 12.0% to 28.0%.

**Conclusions:**

Despite the support from the SUS in Brazil, diagnostic and therapeutic cascades incur additional costs, exacerbating social vulnerability among patients with MDR-TB.

## INTRODUCTION

The World Health Organization (WHO) estimated that 9.9 million people worldwide were diagnosed with tuberculosis (TB) in 2020, equivalent to 127 cases per 100,000 inhabitants. In Brazil, an ascending trend of cases since 2016 is a cause for concern. Currently, Brazil is among the top 30 countries with the highest burden of TB and co-infection with human immunodeficiency virus (HIV)^1^ ^,^ ^2^.

The emergence of the coronavirus disease (COVID-19) pandemic in 2020 presented as an additional challenge in eliminating TB as a public health issue. During this period in Brazil, a national decline in TB incidence, worsening of indicators, such as an increase in loss to follow-up, and reduced utilization of the Xpert^®^MTB/Ultra diagnostic test (Cepheid^©^), was observed, which may be attributed to the overload of the health system and/or issues with data quality^2^ ^,^ ^3^. Furthermore, individuals susceptible to social vulnerability are predictably at a higher risk of infection and disease development, emphasizing that TB fosters the maintenance of impoverishment, contributes to perpetuating social inequality, and presents extensive economic barriers to accessing health services^3^ ^,^ ^4^.

Worldwide, an estimated 150,300 individuals initiated treatment for MDR-TB in 2020. In Brazil, between 2015 and 2020, 7,749 cases of drug-resistant TB (DR-TB) were diagnosed, with 5,377 (69.5%) classified as multidrug-resistant TB (MDR-TB), characterized by resistance to both rifampicin and isoniazid. Notably, the majority of these cases occurred in capital cities (53.0%)^2^. MDR-TB is associated with high mortality rates, particularly among patients co-infected with HIV^4^ ^,^ ^5^ ^-^ ^6^. Currently, eliminating MDR-TB presents a major challenge, as recognized by the WHO, given its critical impact on public health and potential threat it poses to global security and disease prevention. This leads to less efficient therapeutic schemes and is associated with relapses and unfavorable outcomes^7^ ^,^ ^8^ ^,^ ^9^. Additionally, there is limited data on the catastrophic costs incurred by patients with MDR-TB in countries with a high TB burden^10^ ^,^ ^11^ ^,^ ^12^.

In Brazil, access to the diagnosis and treatment of TB is provided at no cost through the Brazilian Unified Health System (SUS, Portuguese), which provides medications, laboratory examinations, and/or necessary imaging examinations. However, additional economic costs incurred by patients and their families can become barriers to treatment adherence.

The total costs stemming from the diagnosis and treatment of MDR-TB, due to the challenges in diagnosis and the longer treatment duration compared to drug-sensitive TB, can lead to significant household budget losses, especially when coupled with income loss^11^ ^,^ ^12^. Notably, the WHO published *The End TB Strategy* in 2016, with a goal to ensure that by 2025, no patient with TB will face catastrophic costs. Our study focused on the financial and economic difficulties resulting from direct and indirect costs, including the cost of incurred when seeking access to healthcare. The percentage of patients (and their families) who experience catastrophic costs due to TB is one of the three main guiding indicators in strategies to eliminate TB^13^ ^,^ ^14^. Specifically, catastrophic costs refer to the total costs incurred by patients undergoing TB treatment that exceeds 20.0% of their annual pre-TB household income^13^. Furthermore, direct costs can be categorized as clinical or non-clinical. Clinical costs include diagnostic tests, doctor’s appointments, examinations, medications, and hospitalization. On the other hand, non-clinical costs include expenses related to transport, food during medical visits, and caretaker fees^15^ ^,^ ^16^. Moreover, indirect costs include gains and losses in productivity related to the intervention, such as the number of days an employee must miss work, resulting in loss in income, revenue, and work hours^15^ ^,^ ^16^. Finally, the cost (or strategy) of coping with the disease includes interventions geared towards mitigating an economy impact, such as borrowing loans, selling of assets, and selling of own merchandise at a reduced value^17^.

Nationally representative studies characterizing TB-related domestic costs have been conducted in several countries, including Vietnam, Uganda, Thailand, Ghana, Indonesia, and Brazil^9^ ^,^ ^10^. However, limited studies have evaluated the catastrophic costs of MDR-TB cases in high-burden countries. Importantly, catastrophic costs may impede patients with MDR-TB from seeking diagnosis and adherence to treatment. MDR-TB is a globally significant disease with deep social determinants linked to the disestablishment and/or absence of effective TB control programs. Thus, analyzing data on catastrophic costs is paramount, as it highlights the need for urgent intervention in the determinants and reducing the occurrence of such costs.

This study aimed to analyze the proportion of catastrophic costs related to the diagnostic and therapeutic pathways for MDR-TB in patients receiving medical care at the Tisiology Outpatient Clinic in the state of Rio de Janeiro. Moreover, we aimed to verify the incidence of impoverishment resulting from diagnostics and treatment plans for MDR-TB at the SUS.

## METHODS

This descriptive prospective study was conducted using a convenience sample of patients with MDR-TB who received medical care at the Tisiology Outpatient Clinic of the Thoracic Diseases Institute of the Federal University of Rio de Janeiro (IDT/UFRJ) between July 2019 and June 2021. 

The inclusion criteria were as follows: a confirmed diagnosis of MDR-TB through genotypic and/or phenotypic testing, recipient of MDR-TB treatment, a minimum of two prior medical consultations before the commencement of this study, willingness to participate, and able to sign an informed consent form. Exclusion criteria included individuals below 18 years of age and incomplete questionnaire responses.

Data collection was performed with a structured cost questionnaire (Supplementary Material 1 **)**, titled “Tool to Estimate Patients’ Costs,” which was developed by the WHO Tuberculosis Control Task Force and adapted for transcultural use by Nunes et al.^10^. The questionnaire uses a quantitative-qualitative approach to assess costs related to TB pre-diagnosis, diagnosis, and treatment. Patients were requested to answer a questionnaire comprising 134 questions. In instances where the patients identified themselves to be incapable of responding, a caretaker, residing in the same household and are familiar with the patient’s routine, will respond to the questionnaire on their behalf. On the same day as the patients’ scheduled doctor appointments, patient interviews were conducted, along with an explanation of the study’s objectives. Written informed consent was obtained from all patients before data collection. The data collection process was performed by the researcher for an average period of 1 h. Moreover, secondary data (medical records) were used to complement patients' financial and clinical information. The collected data covered sociodemographic details, financial information, and clinical costs, including the expenses related to diagnosis, treatment, follow-up, hospitalization, food, and health insurance. Data on direct and indirect costs, as well as the costs of coping with TB were collected to analyze catastrophic costs and impoverishment.

### Data analysis

To calculate the direct prediagnostic and diagnostic costs, this study considered the sum of all administrative costs incurred by the patient, including expenses related to laboratory tests, imaging examinations, medications, travel, food, lodging, as well as any related expenses of companions. These costs were calculated by subtracting any health insurance fees or reimbursements. 

To calculate treatment costs, this study considered the sum of all expenses incurred by the patient, including fees for medical appointments, directly observed treatment (DOT) appointments, medication purchases, follow-up examinations, hospitalization charges, and other applicable expenses. Health insurance reimbursements were deducted from these costs where applicable. Additionally, costs of follow-up appointments, purchase of supplementary medication, cost of follow-up during hospitalization, and caretaker-related costs were included in the calculation. 

Indirect costs were calculated as the sum of total indirect expenses across the pre-diagnosis, diagnosis, and treatment phases, following the guidelines outlined by WHO in the *Tuberculosis Patient Cost Surveys: A Handbook*. The primary parameters of indirect costs were loss of income and the following indirect costs: 1) time spent on consultations, 2) visits to DOT facilities per month, 3) medication, 4) follow-up tests, 5) hospitalization, and 6) expenses associated with other chronic illnesses.

To calculate the work hours during pre-diagnosis and diagnosis/treatment phases, the self-reported incomes before experiencing symptoms were divided by 192 hours/month, following the assumption that 1 week equates to 6 working days with 8 working hours each for a total of 48 working hours/week, and a total of 192 working hours/month . Similarly, to determine to work hours after the illness, we divided the self-reported income post-diagnosis by 192 hours/month. 

Furthermore, the absolute cost of managing TB was calculated. This cost was calculated as the sum of direct and indirect costs (including the cost of coping with TB). All computes costs were then extrapolated to match the projected treatment duration. The catastrophic cost was calculated as the sum of the direct, indirect, and coping costs divided by the total annual household income (annual household income self-reported before the onset of the disease and the estimated annual income based on the equity variation of the goods)^13^ ^,^ ^18^.

Impoverishment was calculated according to the definition of poverty thresholds based on the classifications established by the Brazilian Institute of Geography and Statistics (IBGE, Portuguese)^19^ ^,^ ^20^. The classification “poor” was assigned to individuals whose average household monthly per capita income was below US$ 79,562 for 2019 and US$ 94,5273 for 2020, with annual monetary adjustments. For this calculation, we used the exchange rate between the US dollar and the Brazilian real as of December 01, 2020, at which US$ 1.00 corresponded to R$ 5.2789001^21^. 

For strategies to combat TB, variables, including binary measures, were examined. This study investigated whether families implemented coping strategies while dealing with TB during treatment. The strategies examined included acquisition of a loan or receipt of financial assistance to cover the costs incurred since initiating TB treatment; obligation to sell assets to finance the costs associated with TB treatment; and the patient’s perception of the financial impact of TB on their household since the onset of symptoms.

### Statistical analysis

Descriptive statistics were used to analyze the selected sample data using Microsoft Excel. The distribution of data was observed for skewness to verify the average, mode, and median values. Averages were disregarded when a high degree of data dispersion relative to the average was observed, specifically when the coefficient of variation (CV) exceeded 30.0%. In such instances, medians were employed as central tendency measures to represent the data (Supplementary Material 2).

### Ethical aspects

This research project was registered on the *Plataforma Brasil* (Brazil Platform) and was submitted to the Research Ethics Committee of the Clementino Fraga Filho University Hospital of UFRJ. This project was evaluated and approved under protocol number 3.800.583. This study adhered to all ethical measures in accordance with Resolution 466/12 and the principles of the Declaration of Helsinki.

## RESULTS

A total of 72 patients were interviewed; however, seven were excluded due to incomplete data provided. Consequently, 65 participants were included in this study. Of the participants, 47.7% required a second meeting to further clarify family costs and missing data. The second meeting was prompted by the patients themselves, who expressed lack of knowledge or recollection of certain information, thus enabling them to seek more reliable data.

Table 1 presents the sociodemographic, financial, and clinical characteristics of the participants. The majority (63.1%) of the patients were male. Among the entire cohort, 73.8% were in the continuing treatment stage and 70.8% were HIV-seronegative. In terms of residency, 61.5% of the patients resided in urban areas while 36.9% resided in slums. For education and age, 80.0% attained elementary to high school education, 52.3% were ≤40 years of age, and a predominant portion (40.0%) self-identified as Caucasian. Previous TB treatment was reported by 35.6% of the patients, and 43.1% of them initially sought medical care at a basic health unit (BHU).

**TABLE 1:** Main characteristics of patients with MDR-TB who received medical care in the Reference Tisiology Outpatient Care Clinic of the state of Rio de Janeiro (2019-2021).

	Variable	Total	%	Catastrophic cost		Threshold of Poverty	
				Yes (%)	No (%)	Yes (%)	No (%)
**Sociodemographic**	**Sex**						
	Male	41	63.1	70.7	29.3	17.1	82.9
	Female	24	36.9	79.2	20.8	12.5	87.5
	**Education**						
	Elementary	26	40.0	80.8	19.2	19.2	80.2
	High School	26	40.0	80.8	19.2	19.2	80.2
	Undergraduate	12	18.5	50.0	50.0	0.0	100.0
	Graduate	1	1.5	0.0	100.0	0.0	100.0
	**Age range (years)**						
	≤30	20	30.8	65.0	35.0	10.0	90.0
	31-40	14	21.5	71.4	28.6	14.3	85.7
	41-50	11	16.9	90.9	9.1	9.1	90.9
	>50	20	30.8	75.0	25.0	25.0	75.0
	**Skin color**						
	White	26	40.0	73.1	26.9	3.8	96.2
	Black	19	29.2	84.2	15.8	26.3	73.7
	Brown	20	30.8	65.0	35.0	20.0	80.0
**Financial**	**Household income before TB**						
	No income	8	12.3	37.5	62.5	25.0	75.0
	Up to 1 minimum salary per month	14	21.5	78.6	21.4	7.1	92.9
	Between 1 and 2 minimum salaries per month	23	35.4	78.3	21.7	21.7	78.3
	Between 2 and 3 minimum salaries per month	11	16.9	100.0	0.0	18.2	81.8
	Above 3 minimum salaries per month	9	13.8	55.6	44.4	0.0	100.0
	**Household income after TB**						
	No income	24	36.9	79.2	20.8	25.0	75.0
	Up to 1 minimum salary per month	16	24.6	81.2	18.7	12.5	87.5
	Between 1 and 2 minimum salaries per month	15	23.1	73.3	26.7	13.3	86.7
	Between 2 and 3 minimum salaries per month	4	6.1	75.0	25.0	0.0	100.0
	Above 3 minimum salaries per month	6	9.2	33.3	66.7	0.0	100.0
	**Main breadwinner of the family**						
	Patient	37	56.9	70.3	29.7	16.2	83.8
	Not a patient	28	43.1	78.6	21.4	14.3	85.7
	**Patient works**						
	Yes (Formal, retired, etc.)	26	40.0	57.7	42.3	3.8	96.2
	Yes (Informal)	17	26.1	94.1	5.9	23.5	76.5
	No, unemployed	22	33.8	77.3	22.7	22.7	77.3
	Reason for unemployment related to TB	26	40.0	76.9	23.1	15.4	84.6
	**Participates in government program**						
	Yes	17	26.1	82.3	17.6	23.5	76.5
	No	48	73.9	70.8	29.8	12.5	87.5
	**Location of residency**						
	Urban	40	61.5	72.5	27.5	7.5	92.5
	Community/slum	24	36.9	79.2	20.8	29.2	70.8
	Rural	1	1.5	0.0	100.0	0.0	100.0
	**Stopped working/attends school/does domestic work due to TB**						
	Yes	26	40.0	96.1	3.8	15.4	84.6
	No	39	60.0	58.9	41.0	15.4	84.6
	**Private health insurance**						
	Yes	12	18.5	50.0	50.0	16.7	16.783.3
	No	53	81.5	79.2	20.7	15.1	84.9
**Clinicians**	**Chronic disease**						
	Yes	24	36.9	83.3	16.7	16.7	83.3
	No	41	63.1	68.3	31.7	14.6	85.4
	**Stage of TB treatment**						
	Intensive	17	26.1	94.1	5.9	11.8	88.2
	Continued	48	73.8	66.7	33.3	16.7	83.3
	**HIV serology**						
	Positive	11	16.9	72.7	27.3	9.1	90.9
	Negative	46	70.8	76.1	23.9	19.6	80.4
	Not tested / Ignored	8	12.3	62.5	37.5	0.0	100.0
	**Hospitalization**						
	Yes	28	43.1	75.0	25.0	14.3	85.7
	No	37	56.9	72.9	27.0	16.2	83.8
	**Prior treatment for TB**						
	Yes	23	35.4	78.3	21.7	13.0	87.0
	No	42	64.6	71.4	28.6	16.7	83.3
	**Primary care**						
	Public Hospital	21	32.3	76.2	23.8	19.0	81.0
	Private Hospital/Clinic	15	23.1	66.7	33.3	13.3	86.7
	Health Unit	28	43.1	75.0	25.0	14.3	85.7
	Others	1	1.5	100.0	0.0	0.0	100.0

**TB:** tuberculosis; **HIV:** Human imwmunodeficiency virus.

In terms of income, 35.4% of the patients reported a minimum of one to two salaries, 24.6% reportedly experienced a complete loss of income after TB diagnosis, and 12.3% had no income even before the TB diagnosis. Of the participants, 56.9% reported to be the primary income earner within their families and 73.9% did not receive governmental aid.

Table 2 presents the costs incurred by patients with MDR-TB. Although the mean values are presented, they are not considered in the discussion. Notably, in the identification of the standard deviation, a high degree of data dispersion was observed in relation to the mean, with CV values exceeding 30.0%. The median values for direct, indirect, and total costs were US$ 542.7, US$ 3,825.9, and US$ 4,345.1, respectively. The costs of coping were reported by 47.7% of patients, with a median of US$ 37,886.7.

**TABLE 2:** Costs of treatment to combat MDR-TB in affected patients (2019-2021).

Analyses	Direct cost			Indirect cost	Cost of coping	Total cost
	Patient’s direct cost	Caretaker’s direct cost	Total direct cost			
**Average**	606,1	57,0	**663,1**	5.539,0	52,7	6.254,8
**Standard Deviation**	541,8	70,1	**549,6**	7.599,9	126,6	7.608,4
**Coefficient of variation**	89.4%	123.0%	**82.9%**	137.2%	240.3%	121.6%
**Median**	490,2	24,8	**542,7**	3.825,9	0,0	4.345,1
**Total (US$)**	39.397,8	3.705,1	**43.102,9**	360.036,5	3.425,0	406.564,4

Table 3 presents the patients’ per capita incomes before and after TB diagnosis. Before TB diagnosis, the median per capita incomes of poor and non-poor people were US$ 54.50 and US$ 211.20, respectively. Following the initiation of TB treatment, an approximately 8.0% reduction in per capita income (median, US$ 49.70) was observed among the poor group, as compared to an approximately 10.0% decrease in per capita income (US$ 189.40) among the non-poor group. Impoverishment after the diagnosis and treatment of TB occurred in ten patients with TB (approximately 17.0%). Among the 57 individuals in the non-poor group, eight were already on the verge of poverty before treatment, according to IBGE classifications. 

**TABLE 3:** Household monetary situation and assessment of impoverishment.

Analyses	Income per capita before TB		Income per capita during treatment of TB		Impoverishment
	Poor	Non-poor	Poor	Non-poor	
**Average**	54,1	355,6	51,7	330,6	59,0
**Standard deviation**	20,9	378,4	26,9	363,1	28,4
Coefficient of variation	38.7%	106.4%	52.0%	109.8%	48.1%
**Median**	54,5	211,2	49,7	189,4	71,0
**Total number of patients**	8.0	57.0	18.0	47.0	10.0

### Time spent by the patient in gaining access to health services

On average, the patients experienced a median of two months between the onset of symptoms and the official diagnosis of TB. The nearest healthcare facility providing diagnosis and treatment was approximately 20 min from the patients’ home. Patients typically required approximately three working hours for pre-diagnostic medical consultations and approximately 135 minutes (~2 hours) during visits to receive medications (Table 4).

**TABLE 4:** Time spent by patients during diagnosis and treatment of MDR-TB (n=65).

Time spent by patient with treatment	Months feeling symptoms	How far, in minutes, is the closest health service for diagnosis and treatment?	Duration of pre-diagnosis appointments (hours)	Duration of appointments for receiving medicines (minutes)
**Average**	2,4	35,2	3,2	125,6
**Standard deviation**	1,8	33,9	2,5	126,5
**Coefficient of variation**	77.4%	96.4%	80.0%	100.7%
**Median**	2,0	20,0	3,0	135,0
**Total number of patients**	65.0			

### Total number of families facing catastrophic costs due to illness with MDR-TB

**FIGURE 1:**
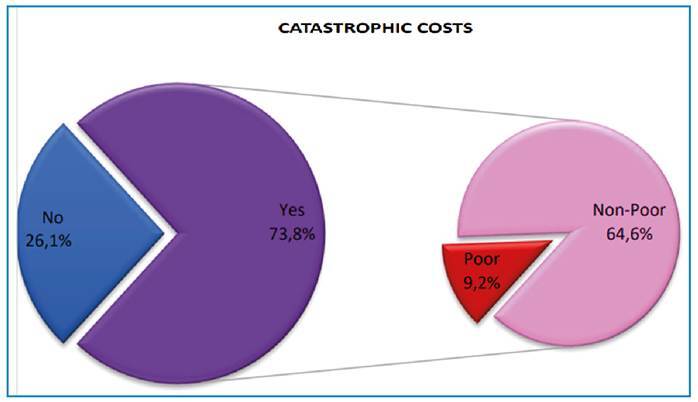
Graph of the percent of families facing catastrophic costs (2019-2021). Poverty was defined according to the threshold established by the Brazilian Institute of Geography and Statistics in 2019 and 2020:

The sum of the direct, indirect, and coping costs resulted in catastrophic costs for 73.8% of the patients. Approximately 64.6% of patients were considered non-poor before the diagnosis of TB (Figure 1). 

Poor people: those with per capita income below the values of US$ 79,562 and US$ 94,5273, respectively.

Non-poor people: those with per capita income above the values of US$ 79,562 and US$ 94,5273, respectively.

## DISCUSSION

This study describes the catastrophic costs incurred by patients with MDR-TB, a topic that is rarely reported in international literature^11^ ^,^ ^22^ ^,^ ^23^.

In this study, a high proportion of patients (73.8%) suffered catastrophic costs, similar to that reported by Rudgard et al.^11^ and Mullerpattan et al.^22^, who assessed these MDR-TB-related costs in Brazil and India, respectively. Notably, our finding is greater than that described by Plesca et al.^23^ (30.0%), who assessed patients with rifampicin-resistant TB in Moldova.

Indirect costs predominantly contributed to the catastrophic cost, which can be justified since the SUS provides free diagnosis and treatment to affected patients. This finding aligns with another study conducted in Brazil^24^, where a strong impact was observed on the indirect costs of patients diagnosed with TB; however, these results differ from those observed in other countries that have no SUS equivalent, such as China, where direct costs were the largest contributor to economic impact^25^.

Similar to most catastrophic cost studies on DR-TB and drug-sensitive TB, our study identified that most patients (52.3%) were affected during their reproductive age (≤ 40 years). This may be attributed to their increased exposure to risk factors and the possibility of losing their jobs or being unable to perform informal work, thereby leading to a partial or total loss of income^26^ ^,^ ^27^.

The coping costs associated with TB were assessed through loan procurement and the sale of personal assists, considering that 47.7% of the patients resorted to these measures, primarily through loan procurement. It is widely acknowledged that families resort to these strategies to avoid health-related ruin or economic shocks, although this may lead to indebtedness. Some participants reported that they had no alternative to such measures or that it was the sole means of coping with the incurred expenses^28^.

Impoverishment following MDR-TB diagnosis was observed in approximately 17.0% of patients who were considered non-poor before diagnosis. This occurred mainly due to the higher reported proportional costs associated with the job loss or an increased need for private treatments and examinations before the TB diagnosis of those considered to be non-poor. In contrast, individuals considered to be poor were already in a highly vulnerable state, with most being unemployed, therefore experiencing less change in their economic status. Wingfield et al.^29^ confirmed that families with the largest economic reduction nearly doubled the chance of incurring catastrophic costs, suggesting that economic shrinkage can be a useful and simple indicator of the risk of catastrophic costs.

This study confirmed that direct and indirect costs cause impoverishment among families affected by MDR-TB. According to Guidoni et al.^30^, the costs generated by TB accentuate the financial strain on patients, particularly those situated in conditions of social vulnerability, strengthening the link between TB and poverty. This is further associated with a decrease in treatment adherence and an increase in the development of DR-TB.

Of the participants included in the study, 73.9% had not received government aid. A study conducted in Brazil by Torrens et al.^31^ described that social protection, such as the Brazilian Family Grant Program (*Bolsa Família*), positively influenced the outcome of TB treatment, with a 5.0% increase in the cure rate observed among patients who received the benefit compared to those who did not. Consequently, public access to these social benefits should be facilitated, and government intervention is needed to overcome the catastrophic scenario of TB.

As hypothesized, among patients with MDR-TB, treatment history of previous TB treatment was noted in 35.4% of patients. It is widely acknowledged that cases of TB retreatment tend to present with less favorable outcomes, culminating in treatment interruption and new means of drug resistance, leading to complications in TB control and higher costs of treatment compared to new cases. This is further substantiated by the analysis of the Information System for Special Tuberculosis Treatments, which indicates that the majority (74.8%) of cases involved new cases of TBDR, followed by cases of reentry after treatment abandonment (13.1%), and cases of treatment failure (7.5%)^32^ ^,^ ^33^.

The high proportion (55.4%) of patients who sought initial care in both public and private hospital units was similar to that reported by Maior et al.^34^ (69.0%). This may be attributed to patient disinformation and greater resources in hospitals, which can provide swift diagnoses. Moreover, the scenario may vary if a public SUS offers on-demand medical care. Indeed, patients frequently seek medical attention but are not accommodated due to a lack of scheduling, compelling them to seek alternatives in emergency care in hospital units. A similar finding was cited by Amarante et al.^35^, suggesting that emergency health units are commonly used as diagnostic units because of larger technological structures, swiftness, and agility. Therefore, they have a greater ability to resolve health problems. Notably, BHUs remain as treatment “control” units.

On average, the onset of symptoms to correct diagnosis and referral to reference outpatient clinics takes approximately two months. Similarly, treatment waiting times are prolonged, with an average wait of two hours to receive the medications. This delay in diagnosis and treatment is supported by patient reports of diagnostic errors and the fragility of health systems, which indicates the need for effective professional training regarding the appropriate handling of patients suspected with TB. The delays in the provision of medical care and the increasing transmission of MDR-TB in the community result in additional expenses and a greater burden to income. The current situation in Brazil, exacerbated by the pandemic, presents multiple challenges, such as the decreased value of governmental aid that cannot meet substantial needs and increased unemployment due to lack in investments, which increases the possibility of catastrophic costs. The World Bank of Brazil reported that the COVID-19 pandemic has exposed Brazil to unprecedented sanitary and economic challenges, with various uncertainties in its macroeconomic political structure^36^.

Therefore, it is crucial to establish measures to reduce costs associated with TB. These initiatives may include professional training in educational institutions (medical and nursing schools) to ensure the prompt diagnosis of diseases at different levels of healthcare, implementation of alternative opening hours at BHUs, reduction of waiting time at BHUs, and improvement of healthcare infrastructures. Considering that efforts to achieve an early diagnosis can impact direct and indirect costs, the financial burden on families can be reduced^24^. 

This study has some limitations. First, due to the nature of this report, utilizing a questionnaire, there was a potential for memory bias, given that the majority (73.9%) of the participants received anti-TB treatment for over two months. Second, over or underestimation of expenses was possible due to the self-reporting of participants. Third, as this was a single-center study with a convenience sample, its generalizability may be limited; however, this study was carried out in one of the three state reference centers for MDR-TB in Rio de Janeiro; thus, the results may be representative of the state of Rio de Janeiro. Convenience sampling was the most operational method in the collection of data because patient access was limited and further exacerbated by the pandemic. In this context, to mitigate memory bias, we gathered medical and social service records to supplement the data. Furthermore, a second patient meeting was scheduled when necessary to provide patients with the necessary time and resources to collect the required information.

## CONCLUSION

Despite the availability and cost-free diagnostics and treatment plans for MDR-TB in Brazil through the SUS, patients and their families continue to grapple with severe economic impacts, such as catastrophic costs. These financial burdens can contribute to the discontinuation of treatment, leading to the emergence of new drug-resistant TB strains. Therefore, social policies supporting the patients and their families must be implemented and maintained to eradicate the disease.
