# Supplementary material for: Analysis of catastrophic costs incurred by patients with multidrug-resistant tuberculosis in an outpatient clinic in the state of Rio de Janeiro
Source: Rev Soc Bras Med Trop. 2023 Oct 30;56:e0148-2023. doi: 10.1590/0037-8682-0148-2023 (PMC10615334; doi:10.1590/0037-8682-0148-2023)
Supplement: Supplementary file 1 [file 1678-9849-rsbmt-56-e0148-2023-supp1.pdf]

COST QUESTIONNAIRE

Name:____ **Medical Record:**__________

Interview Date:_____/______/______ **Interview Location: :**____________

PART I - GENERAL INFORMATION

**1. Sex:** 1.Male 2. Female	**2.Age:**__________	**3. Outcome: TB**
**4. Kind of TB:**	**MDR TB**	
**5. Total duration of planned treatment:**	1. (6 months) 2. (8 months) 3. Others ________ months	
**6. Treatment regimen:**	1. Category I (Pulmonary / new) 2. Category II (retreatment)<br>3. Category III (New or Extra-pulmonary) 4. Category V (chronic, MDR)	
**7. Currently at what stage of TB treatment?**	1. Intensive 2. Continued	
**8. Who is being interviewed?**	1. Patient 2. Responsible 3. Others ________	
**9. TB treatment start date:** _____/_____/______	**10. HIV serology:** 1. Positive 2. Negative 3. Not tested 4. Ignored	
**11. Investigation start date:** ______/______/______		

PART II - PREVIOUS TB TREATMENT

**12. Have you received treatment for TB before?** ***(If not go to part III)***	1. Yes (month/year) _______/_______ 2. No
**13. If yes: You have completed previous TB treatment?**	1. Yes 2. No
**14. If not, why not?**	1. Lack of money for treatment costs 2. Drug side effects 3. Change 4. Distance from service<br>5. Others (to specify):____

PART III - DELAY, PRE-DIAGNOSIS AND DIAGNOSIS COSTS

15. What symptoms did you experience that led you to seek treatment for your current illness? How long did you experience these symptoms before seeking treatment??

1. Cough yes □ no □ ________ months 2. night sweats yes □ no □ ________ months
3. Coughing up blood yes □ no □ ________ months 4. Weight loss yes □ no □ ________ months
5. Others / to specify yes □ no □ ________ months /____

16. Did you seek treatment or counseling for these symptoms in any of the follow-ups? Check everything that applies. Where did you go first? Circle the first place of treatment.

1. Local hospital yes □ no □ 5. Pharmacy or drug store yes □ no □
2. TB control program yes □ no □ 6. Phytotherapy yes □ no □
3. Health Unit yes □ no □ 7. Private hospital/clinic yes □ no □
4. Mission hospital / campaign/regional yes □ no □ 8. Other / to specify yes □ no □ / __________________________

**17. Have you ever visited a traditional healer?**	1. Yes 2. No

If another option other than public service was chosen in question 16.

18. Why didn't you go to a public health facility, such as a health facility or hospital, when you realized you were sick? Circle the most applicable.

1. Distance from service
2. Too expensive
3. Long waiting time
4. Lack of available services
5. Lack of confidence in public services offered
6. Personal beliefs
7. No drugs available
8. Others (to specify):____

19. How far is the nearest health facility to:

a) Diagnosis and Treatment: _______hours walking _______hours transport Others:_________

b) Treatment Only: _______hours walking _______hours transport Others:_________

20. How much did you spend for each of these visits before you were diagnosed, including the visit when you actually received your TB diagnosis? On the next page, for all that do not apply, mark N/A; Fill in one line per visit.

	**Provider** (where the patient sought treatment or advice).	**Total time spent per visit** (in hours, including travel time).	**Administrative costs**	**Testing Costs** (for bacilloscopy or others except x-ray).	**x-ray costs** (includes sending an x-ray to the radiologist, travel and fees).	**Medication costs**	**Travel costs** (all returns)	**Food costs** (total)	**Accommodation Costs** (total)	**Sub-Total costs per visit**	**Refund insurance. Subsistence allowance** (If yes: quantity, if not N/A)
**Visit 1**											
**Visit 2**											
**Visit 3**											
**Visit 4**											
**Visit 5**											
**Visit 6**											
**Visit 7**											
**Visit 8**											
**Visit 9**											
**Visit 10**											
**Total**											
**Total Direct Costs of Pre-diagnosis and Diagnosis (sum of sub-totals) less insurance =**									**Currency name:**		

PART IV - TB TREATMENT COSTS	
**21. Where do you currently take your TB drugs?** 1. Health Unit/Hospital 2. Residence 3. Community 4. Workplace 5. Dispensary/Pharmacy	
**22. How many times a week do you go to the location to take your medications?**	□ 3 times □ 5 times □ 6 times □ Others
**23. How long does it take you to get there?** (one way)	____ hours walking ____ hours with transport Others:__________
**24. How long does one of these visits last on average, including time on the road and waiting? (total travel time).**	_________ hours
**25. From your home to the TDO location, how much does it cost if you take transport? (both ways).**	US$________
**26. How much do you spend on food (on the road, while waiting and for lunch)?**	US$________
**PART V - COSTS RELATED TO DISTRIBUTION OF TB MEDICINES, WHERE DRUGS ARE CURRENTLY DISTRIBUTED.**	
**27. How often do you travel to the health center/hospital to pick up your TB drugs?**	________times/month
**28. How long does it take you to get there (one way)**	____ hours walking ___ hours with transport _____ Others
**29. How long does one of these visits take on average, including time on the road and waiting time? (total travel time)**	__________ hours
**30. From your home to the service, how much does it cost if you take transport? (both directions)**	US$____________
**31. If you go to a service to pick up your medication, how much do you spend on food that day? (about the route, while waiting, lunch etc)**	US$____________
**32. a) Do you have to pay administrative fees when you pick up your TB drugs? (If no, go to 33. b) If YES, how much?**	1. Yes 2.No US$ ___________
**33. a) Do you have any accommodation costs when you pick up your TB drugs? (If not, go to 42). b) If YES, how much?**	1. Yes 2.No US$ ___________
**PART VI – COST OF MEDICAL CONSULTATIONS**	
**34- How often do you travel to the health center/hospital for medical appointments?**	________ times/month
**35. How long does it take you to get there (one way)**	____ hours walking ___ hours with transport _____ Others
**36. How long does one of these visits take on average, including time on the road and waiting time? (total travel time)**	__________ hours
**37. From your home to the service, how much does it cost if you take transport? (both directions)**	US$___________________
**38. If you go to a service to have a consultation, how much do you spend on food that day? (about the route, while waiting, lunch etc)**	US$____________
**39. a) Do you have to pay administrative fees when carrying out consultations? (If not, go to 33). b) If YES, how much?**	US$________
**40. a) Do you have any accommodation costs when you go to the appointments? (If not, go to 42).** **b) If YES, how much?**	1. Yes 2. No
**41. Number of consultations carried out so far?**	________ consultations

PART VII - COSTS RELATED TO FOLLOW-UP OF TESTS TB TREATMENT	
**42. Have you ever had to go to the health services, in addition to your regular visits to follow up on control tests since starting treatment? (If not, go to 47)**	1. Yes 2. No
**43. If yes, how many times?**	______Times
**4. If yes, have you ever had to pay any additional costs during the treatment period?**	1. Yes 2. No
**45. If so, what kind of cost and how much?**<br>**Fees _______________ Sputum exam___________X-ray______________ TB drugs _______ Drugs______________ Others ___________**	Total:_________
**46. How long does one of these routine visits take on average, including time on the road, waiting time, and testing? (total for the entire route)**	________ hours

PART VIII - CUSTODY COSTS/ FOLLOW-UP	
**47. Does any family member/friend/professional support accompany or accompany you on some visits or to your place of treatment to pick up your TB drugs? (If not, go to 51).**	1. Yes 2. No
**48. If YES, on how many visits did your family member/friend/professional support accompany you or go to your place of treatment? Report pre-diagnosis/diagnosis visits, and treatment separately.**<br>Complete with data record:<br>Costs per visit Pre-diagnosis / diagnosis: Transport______ Food______ Accommodation _________<br>Costs during treatment per visit: Transport _____ Food______ Accommodation __________	_____ Pre-diagnosis/diagnosis visits<br>_____ Treatment visits<br>Diagnosis total:__________________<br>Total treatment:_________________
**49. Do you pay your family member/friend/professional support per day for follow-up?**	1. Yes, US$_____________day 2. No
**50. Why did someone accompany you?**	1. Distance 2. Security 3. Administrative Barriers 4. Too Sick to Travel Alone<br>5. Was requested for treatment 6. Others (to specify)____

PART IX – HOSPITALIZATION	
**51. Have you ever been hospitalized before or during your TB treatment? (If No, go to 63).**	1. Yes 2. No
**52. If YES, how many days in total were you in the hospital?**	______Days
**53. How much did you pay at the hospital during your stay?**<br>Hospital administration fees: ___________ Sheets/linens:____________ Medicines: _______________<br>Food (not provided by the hospital): _________ Transport (return):__________ Tests:___________ Others: ____________________________	Total: US$__________
**54. Was any family member/friend with you at the hospital? (If not, go to question 58).**	1. Yes 2. No
**55. If YES, how many days did he/she stay with you? (slept there)?**	_______Days
**56. Were there any extra costs for your family/friend for the hospital stay?**	1. Yes 2. No

Accommodation (hospital or Others): ___________ Food: ____________ Transportation: ____________Other: _______________	Total costs:___________
**57. How much do your friends/family usually earn per day?**	1. Win US$__________ 2. don't win
**58. Did any other family members/friends visit you while you were in the hospital? (If not, go to 63).**	1. Yes 2. No
**59. If yes, how many people visited you?**	______ people at a time
**60. How many times did they visit you?**	Total number of visits: __________
**61. Spending per person: Accommodation: __________ Meals: __________ Transport: __________ Others:________________**	Total costs per person: US$________
**62. How long did the visits last including travel time?**	__________ hours
**PART X - OTHER COSTS: FOOD SUPPLEMENTS**	
**63. Do you buy any supplements to your diet because of your TB illness, for example vitamins, meats, energy drinks, juices, fruits or medicine? (If not, go to 66).**	1. Yes 2. No
**64. If YES: What type of items?**	1. Fruits 2. Beverages 3. Vitamins/Herbs 4. Meat 5. Others (to specify):_______________________
**65. How much did you spend on these items in the last month or so?**	US$_____________
**PART XI - OTHER DISEASES**	
**66. Do you have any chronic illnesses for which you receive treatment? (If not, go to 72).**	1. Yes 2. No
**67. If yes, which ones?**	____
**68. Are there any additional costs for you because of this illness in addition to the costs you have already mentioned? (If not, go to 72).**	1. Yes 2. No
**69. If YES, how many are these additional costs on average per month?**<br><br>Tests: _________ Medicines: _________ Transportation: _________ Food: _________ Others: _________	Total: _________
**70. How much did you spend on health care, on average, per month, before you became ill with TB?**	US$_________
**71. How much do you spend on health care on average per month now?**	US$_________
**PART XII – SAFE**	
**72. Do you have any kind of private or government health insurance/medical insurance? (If not, turn to 76).**	1. Yes 2. No
**73. If YES, what type?**	1. Monthly health subsidy 2. Donation 3. Family or community funds 4. Health insurance/Supplementary 5. Others (to specify)
**74. Have you received reimbursement for all costs related to your illness? Cross check with question 20 (table on pre-diagnosis and diagnostic costs)-(If No, go to 76).**	1. Yes 2. No
**75. How much did you receive as a refund?**	US$___________
**PART XIII - FINANCED COSTS**	
**76. Did you borrow any money to cover the costs of being ill? (If not, go to question 80).**	1. Yes 2. No
**77. If YES: How much did you borrow?**	US$____________
**78. Who did you borrow from?**	1. Family 2. Neighbors 3. Private Bank 4. Cooperative 5. Others (to specify):________________________
**79. What is the loan interest rate?**	1. less than 5%. 2. 5% to 10% 3. More than 10% 4. I don't pay any interest 5. I won't have to pay the money

**80. Have you ever sold any part of your property to finance the cost of your illness? (If not, go to 84)**	1. Yes 2. No
**81. If YES, what did you sell?** Circle the most appropriate:	1. Rural property 2. Livestock 3. Transport / vehicle 4. Household item 5. Rural products<br>6. Others (to specify):_________________________ _
**82. What is the estimated market value of the property or other assets you sold?**	**US$**____________
**83. How much did you earn from the sale of your property or other assets that you sold?**	**US$**____________

PART XIV - SOCIECONOMIC INFORMATION, INDIVIDUAL SITUATION AND INCOME

**84. Who is the family's main income earner?**	1. Patient 2. Wife/mother 3. Husband/father 4. Relatives 5.Son/daughter<br>6. Others (to specify)____
**85. What is the patient's highest level of education?**	**1**. Illiterate **2**. Elementary (1º a 9º year) **3** Average (1º ao 3º year) **4**. Graduation / Postgraduate
**86. What is the highest level of education main income provider?**	**1**. Illiterate **2**. Elementary (1º a 9º year) **3.** Average (1º ao 3º year) **4**. Graduation / Postgraduate
**87. What is the highest level of education of the head of household?**	**1**. Illiterate **2**. Elementary (1º a 9º year) **3.** Average (1º ao 3º year) **4**. Graduation / Postgraduate
**88. What is the highest level of education of the head of household's spouse?** If more than one spouse, choose highest level of education.	**1**. Illiterate **2**. Elementary (1º a 9º year) **3.** Average (1º ao 3º year) **4**. Graduation / Postgraduate

89. Are you currently formally employed?

1. Yes, formal job (go to 92)
2. No, informal work/ Unemployed (go to 90)
3. On sick leave (turn to 90)
4. Retired (go to 90)
5. School, university (go to 98)
6. Housework (turn to 92)
7. Others (to specify) _______________________

**90. Is the reason for not working related to the TB illness? (if not, go to 92)**	1. Yes 2. No
**91. If YES, when was the last time you were working?** (month/year)	______/_______
**92. How you are/were usually paid?**	1. In cash 2. In goods 3. In cash and goods 4. Unpaid 5. Salary by transfer/deposit 6. Others

93. What was your estimated personal income per month for the family BEFORE before your TB illness? (includes welfare, disability, or other social support):

1. Up to 1 (one) salary per month
2. Between 1 (one) and 2 (two) salaries per month
3. Between 2 (two) and 3 (three) salaries per month
4. More than 3 (three) salaries per month
5. Does not earn

94. What is your estimated personal income per month for the family NOW? (includes welfare, disability, or other social support)

1. Up to 1 (one) salary per month
2. Between 1 (one) and 2 (two) salaries per month
3. Between 2 (two) and 3 (three) salaries per month
4. More than 3 (three) salaries per month
5. Does not earn

**95. If answer 93 differs from 94. Is the change related to the TB illness?**	1. Yes 2. No
**96. Have you ever stopped working / going to school / doing housework because of TB? (If not, turn to 98).**	1. Yes 2. No
**97. If YES, for how long?**	1. Less than 1 month 2. 1 month 3. 2-3 months 4. 4-5 months 5. More than 6 months
**98. Has anyone ever stayed home specifically to take care of you?**	1. Yes 2. No
**99. If so: for how long?**	______________weeks
**100. Has anyone ever quit their income-generating job to stay home and take care of you?**	1. Yes 2. No

**101. What was the regularity of your work before you fell ill?**	1. Throughout the year 2. Season/part of the year 3. Working day 4. Other________________
**102. You had to change jobs when you got sick?**	1. Yes 2. No
**103. What is your main occupation? Tick all that apply as per question 89.**<br>1. Sales/Service 3. Households 5. Combination (to specify)________________ 7. Health<br>2. Agriculture 4. Production/construction 6.Others (to specify)______________________ 8. Education	
**104. How many hours did you work on average per day before becoming ill with tuberculosis?**	___________hours
**105. How many hours do you work, on average, per day now?**	___________hours
(If the answer to 104 differs from the answer to 105): **106. Is the change related to the TB disease?**	1. Yes 2. No
(If answer 104 differs from answer 105):<br>**107.a) Is anyone doing the work you used to do?**	1. daughter 2. son 3. spouse 4. friend 5. nobody 6. other family
**108. Do you have children under school age? (If NO, go to 112).**	1. Yes 2. No
**109. Do all your school-age children attend school regularly? (If YES, go to 111)**	1. Yes 2. No
**110. If NO: Why NOT? Circle the most suitable**<br>**1. Need to help around the house** **2. No money for school fees** **5. Others (to specify):** ____<br>**3. Also sick** **4. You have to work to earn income**	
**111. Do any of your children under school age work to finance the costs of becoming ill with TB?**	1. Yes 2. No
**112. Do you pay/did you pay someone to do housework at your home? (if not, go to 115)**	1. Yes 2. No
**113. How much do/did you pay while you are/were sick?**	US$______________________
**114. When did you pay BEFORE the illness?**	US$______________________
**115. Are you financially independent?**	1. Yes 2. No
**116. Has having TB disease affected your social or private life in any way? (If NO, go to 118).**<br>**1.** No 3. Job loss 5. Separated from spouse/partner 7. Sick child<br>2. Divorce 4. Dropped out of school 6. Disturbed sex life 8. Others (to specify):_____________________________________	
**117. If yes, did having this result in a financial burden?**	1. Yes 2. No
**118. What color is your skin?** 1. White 2. Black 3. Yellow 4. Brown 5. Indigenous	**118. a. What is your religion?**____________________________________
**PART XV - FAMILY INCOME AND EXPENSES**	
**119. Are you part of any Government Social Program? Or someone in your family? (if NO, go to 122)**	1. Yes 2. No
**120. If YES, which one/which ones?** (circle more than one if applicable)	1. Family scholarship

	2. My Home My Life<br>3. Continuous Payment Benefit (BPC – 01 monthly minimum wage for the Elderly and/or physically, mentally or intellectually disabled)<br>4. Bolsa Verde (family in extreme poverty living in a preservation area)<br>5. Child Labor Eradication Program (PETI – from 25 to 40 reais per child under 16)
**121. What is the total value of these benefits?**	US$________________________________
**122. Do you or anyone in your family receive any other social support?**	1. Basic Basket 2. Social Voucher 3. Other (to specify:_____________)
**123. Which place helps you with this social support?**____	
**124. How much do you estimate your average household income per month BEFORE you got sick with TB? (All people in the household, including patient, government assistance or other social support).**<br>1. Patient income_________________ 3. Pension payments____________________ 5. Other: ______________<br>2. Income of household residents_________________ 4. Government assistance ____________________ **TOTAL:**______________	
**125. How much do you estimate the average income for your household per month RIGHT NOW?**<br>1. Patient income_________________ 3. Pension payments____________________ 5. Other: ______________<br>2. Income of household residents_________________ 4. Government assistance ____________________ **TOTAL:**______________	
**126. How many people regularly sleep in your house? (including the patient)** (If the patient lives alone, go to question 130 and replace the word "house" with **"you").**_______________________	
**127. How many people in your household work? (including patient)**	_________________________
**128. Besides yourself, does anyone in your family receive treatment for tuberculosis?** (If not, turn to 130).	1. Yes 2.No
**129. If YES, how many?**	_____________________________
**130. How much food did your household consume per month, on average, BEFORE the TB disease (value in money)?**	Calculate the value:________________________________
**131. If home production BEFORE (cash value):**	Calculate the value:________________________________
**132. How much food does your household consume NOW each month on average (for the same number of people)?**	Calculate the value:________________________________
**133. If home production NOW (cash value):**	Calculate the value:________________________________
**134. If the answer to 133 differs from 131: Has the amount of food consumed per month changed due to TB disease?**	1. Yes 2. No
**PART XVI - INDICADORES SOCIOECONÔMICOS**	
**135. What is your energy supply?**	1. Own connection 2. Shared connection 3. None
**136. What is your source of drinking water?**<br>1. Rainwater 2. Lakes/Lagoa/River 3. Public Supply 4. Private Supply/Cistern 5. Piped Water 6. Mineral Water	

**137. What type of toilet facility is available?**	1. Not available/bushes/fields 2. Shared toilet 3. Own toilet 4. Septic tank
**138. How many rooms are there in your house?**	1. (1 room) 2. (2 rooms) 3. (3 rooms) 4. (4 or more rooms)
**139. Current place of residence?**	1. Urban 2. Clustered (community/favela) 3. Rural 4. Other (to specify)
**140. Do you own the house or residence where you live?**	1. Yes 2. No

141. You have... Includes standard goods adapted for the country's Health and Demographic Survey.

1. Cell phone	4. Bicycle	7. Refrigerator	10. Stove
2. Washing machine	5. Property (quantity)__________	8. Television	11. Radio (including integrated into another type of device)
3. Motorcycle	6. Car	9. Microwave	12. Computer with internet access

**142. If the government could provide you with any service to ease the burden of TB on you and your family, what would you prefer to receive? Choose one of the following options:**	1. Transport voucher 3. More efficient service<br>2. Meal allowance 4. Other (to specify):_______________

We would like to know the cost of becoming ill with TB on your family's well-being; that is, we would like to place the value of illness caused by TB, including pain and suffering.

So we would like to know how much it would be worth it to you if you could prevent TB in the first place. Note that we don't ask what you can currently afford, but what you would be willing to pay if you had an unlimited amount of money.

143. How much would you be willing to pay to not get sick with TB in the first place?

1. Up to 1(one) salary per month 2. Between 1(one) and 2(two) salaries per month 3. More than 3(three) salaries per month 4. Other (to specify)____________________

Thanks for your cooperation! Would you like to ask or say something?

Interviewer comments:

Date, Interviewer's signature: ________/________/_____________ ______________________________
