# Supplementary material for: Analysis of catastrophic costs incurred by patients with multidrug-resistant tuberculosis in an outpatient clinic in the state of Rio de Janeiro
Source: Rev Soc Bras Med Trop. 2023 Oct 30;56:e0148-2023. doi: 10.1590/0037-8682-0148-2023 (PMC10615334; doi:10.1590/0037-8682-0148-2023)
Supplement: Supplementary file 2 [file 1678-9849-rsbmt-56-e0148-2023-supp2.pdf]

Medical Record	Annual Family Income by IBGE Assets	Annual family income b	Total Family Income	Total Direct Costs of Treatment	Total Indirect Treatmen	Total Coping Costs	Total Diagnostic Cost	Total Cost Treatment + Diagnosis+Coping	Lost Income TB Treatment	Percentage
531493	4280,04	12600,00	16880,04	2010,60	29452,34	1000,00	65,21	32528,16	1,93	1,93
529478	8891,88	38400,00	47291,88	2690,00	32175,00	100,00	212,50	35177,50	0,74	0,74
531504	4280,04	16200,00	20480,04	359,50	23639,06	200,00	63,28	24261,84	1,18	1,18
531949	6472,68	31800,00	38272,68	1970,10	6045,31	0,00	118,70	8134,11	0,21	0,21
533140	8891,88	50292,00	59183,88	180,00	20104,65	300,00	191,67	20776,32	0,35	0,35
515631	4280,04	22800,00	27080,04	1029,20	-4332,81	0,00	10,42	-3293,20	-0,12	-0,12
162993	22042,08	216000,00	238042,08	8380,50	7171,88	0,00	122,50	15674,88	0,07	0,07
526887	6472,68	31200,00	37672,68	1408,20	78015,10	40,00	18,75	79482,05	2,11	2,11
531736	6472,68	24000,00	30472,68	448,30	19395,23	0,00	55,45	19898,98	0,65	0,65
533073	8891,88	42000,00	50891,88	3977,50	25077,52	100,00	719,20	29874,22	0,59	0,59
534602	6472,68	24000,00	30472,68	3365,60	1184,90	400,00	39,30	4989,80	0,16	0,16
529022	22042,08	182400,00	204442,08	1316,00	30538,96	0,00	252,50	32107,46	0,16	0,16
530143	8891,88	62400,00	71291,88	2709,30	97107,81	0,00	103,13	99920,24	1,40	1,40
592748	8891,88	60000,00	68891,88	3234,20	8542,97	0,00	802,50	12579,67	0,18	0,18
534022	6472,68	24000,00	30472,68	770,10	26755,21	500,00	10,42	28035,73	0,92	0,92
709364	6472,68	25200,00	31672,68	2765,20	34392,71	120,00	723,75	38001,66	1,20	1,20
715565	9088,32	36000,00	45088,32	1282,00	4476,56	40,00	147,00	5945,56	0,13	0,13
711886	6472,68	24000,00	30472,68	4200,00	375,00	0,00	53,30	4628,30	0,15	0,15
717712	8891,88	48000,00	56891,88	2061,90	8825,00	0,00	126,68	11013,58	0,19	0,19
707342	8891,88	62400,00	71291,88	9860,00	65425,00	500,00	211,10	75996,10	1,07	1,07
525454	4280,04	16800,00	21080,04	533,40	3514,58	0,00	54,88	4102,87	0,19	0,19
525455	4280,04	21600,00	25880,04	3302,60	28265,63	50,00	15,63	31633,85	1,22	1,22
723009	4280,04	20400,00	24680,04	2200,90	159,38	500,00	39,93	2900,20	0,12	0,12
528759	8891,88	36000,00	44891,88	4725,90	93604,17	0,00	53,13	98383,20	2,19	2,19
529354	4280,04	21600,00	25880,04	3624,00	3670,31	1000,00	190,00	8484,31	0,33	0,33
727679	4280,04	13200,00	17480,04	8209,60	4357,03	600,00	1849,38	15016,01	0,86	0,86
528150	4280,04	17760,00	22040,04	3955,10	23125,47	100,00	56,51	27237,08	1,24	1,24
534647	6472,68	30000,00	36472,68	819,80	22178,91	0,00	375,31	23374,02	0,64	0,64
718860	12429,48	84000,00	96429,48	6740,00	-15828,13	0,00	2160,00	-6928,13	-0,07	-0,07
534022	8891,88	38160,00	47051,88	10053,30	36242,73	2600,00	237,50	49133,53	1,04	1,04
527935	6472,68	26400,00	32872,68	2763,00	50327,34	0,00	1044,40	54134,74	1,65	1,65
527936	8891,88	36000,00	44891,88	5679,45	44093,75	0,00	93,75	49866,95	1,11	1,11
532574	4280,04	15600,00	19880,04	9604,80	20712,50	4000,00	2654,13	36971,43	1,86	1,86
540114	8891,88	67200,00	76091,88	5952,70	58141,13	2000,00	374,38	66468,20	0,87	0,87
540113	12429,48	84000,00	96429,48	1959,60	51187,50	0,00	503,17	53650,27	0,56	0,56
727929	6472,68	26400,00	32872,68	6019,60	24947,66	0,00	0,00	30967,26	0,94	0,94
734810	12429,48	72000,00	84429,48	12225,00	67050,00	0,00	420,00	79695,00	0,94	0,94
727407	8891,88	57600,00	66491,88	2676,00	29406,25	0,00	264,06	32346,31	0,49	0,49
473394	4280,04	15600,00	19880,04	3720,00	7021,59	0,00	821,58	11563,18	0,58	0,58
702449	8891,88	40140,00	49031,88	4101,20	18196,30	150,00	141,18	22588,68	0,46	0,46
728290	12429,48	75240,00	87669,48	1033,40	45699,32	0,00	517,85	47250,57	0,54	0,54
730009	15576,60	144000,00	159576,60	7840,10	8968,13	0,00	310,00	17118,23	0,11	0,11
727545	8891,88	58200,00	67091,88	2252,80	-7381,64	0,00	298,83	-4830,02	-0,07	-0,07
705225	6472,68	30000,00	36472,68	4932,00	17887,50	30,00	88,08	22937,58	0,63	0,63
702396	4280,04	4104,00	8384,04	864,20	10636,59	100,00	26,25	11627,04	1,39	1,39
736115	6472,68	24540,00	31012,68	760,00	19817,81	0,00	271,98	20849,80	0,67	0,67
730283	8891,88	42540,00	51431,88	840,00	100942,62	850,00	95,13	102727,74	2,00	2,00
720433	4280,04	12540,00	16820,04	1168,80	9690,74	0,00	10,89	10870,43	0,65	0,65
733325	6472,68	31200,00	37672,68	1604,00	22647,66	200,00	120,83	24572,49	0,65	0,65
728844	6472,68	25080,00	31552,68	5081,00	3202,27	400,00	10,89	8694,15	0,28	0,28
735071	4280,04	3600,00	7880,04	693,00	7375,83	30,00	8,10	8106,93	1,03	1,03
521335	4280,04	3600,00	7880,04	1900,00	386,72	1500,00	0,00	3786,72	0,48	0,48
536293	4280,04	14940,00	19220,04	1440,00	39654,38	200,00	10,89	41305,26	2,15	2,15
723258	6472,68	25200,00	31672,68	2160,90	9612,76	0,00	103,33	11876,99	0,37	0,37
728401	4280,04	18000,00	22280,04	162,00	2285,16	400,00	32,81	2879,97	0,13	0,13
735294	22042,08	240000,00	262042,08	315,90	245507,81	0,00	156,25	245979,96	0,94	0,94
735295	8891,88	43200,00	52091,88	2997,60	21778,13	0,00	723,13	25498,85	0,49	0,49
735296	6472,68	24960,00	31432,68	2438,20	2946,67	20,00	40,00	5444,87	0,17	0,17
735297	4280,04	14400,00	18680,04	2414,60	43200,00	50,00	512,50	46177,10	2,47	2,47
526399	4280,04	13200,00	17480,04	0,00	309,38	0,00	664,58	973,96	0,06	0,06
526398	8891,88	64800,00	73691,88	3401,40	150768,75	0,00	466,25	154636,40	2,10	2,10
471527	8891,88	34800,00	43691,88	3704,60	22731,25	0,00	105,21	26541,06	0,61	0,61
471526	8891,88	58800,00	67691,88	3299,10	6660,94	0,00	377,33	10337,37	0,15	0,15
530021	6472,68	30000,00	36472,68	3744,00	5683,59	0,00	250,19	9677,78	0,27	0,27
530020	8891,88	42000,00	50891,88	2869,60	19057,81	0,00	172,50	22099,91	0,43	0,43
